# Transcriptomic Analysis of Arachidonic Acid Pathway Genes Provides Mechanistic Insight into Multi-Organ Inflammatory and Vascular Diseases

**DOI:** 10.3390/genes15070954

**Published:** 2024-07-20

**Authors:** Vaishnavi Aradhyula, Joshua D. Breidenbach, Bella Z. Khatib-Shahidi, Julia N. Slogar, Sonia A. Eyong, Dhilhani Faleel, Prabhatchandra Dube, Rajesh Gupta, Samer J. Khouri, Steven T. Haller, David J. Kennedy

**Affiliations:** 1 Department of Medicine, The University of Toledo College of Medicine and Life Sciences, Toledo, OH 43614, USA; 2 Biochemistry and Biotechnology Group, Bioscience Division, Los Alamos National Laboratory, Los Alamos, NM 87545, USA

**Keywords:** arachidonic acid, eicosanoids, transcriptomics, vascular inflammation, inflammatory vascular remodeling

## Abstract

Arachidonic acid (AA) metabolites have been associated with several diseases across various organ systems, including the cardiovascular, pulmonary, and renal systems. Lipid mediators generated from AA oxidation have been studied to control macrophages, T-cells, cytokines, and fibroblasts, and regulate inflammatory mediators that induce vascular remodeling and dysfunction. AA is metabolized by cyclooxygenase (COX), lipoxygenase (LOX), and cytochrome P450 (CYP) to generate anti-inflammatory, pro-inflammatory, and pro-resolutory oxidized lipids. As comorbid states such as diabetes, hypertension, and obesity become more prevalent in cardiovascular disease, studying the expression of AA pathway genes and their association with these diseases can provide unique pathophysiological insights. In addition, the AA pathway of oxidized lipids exhibits diverse functions across different organ systems, where a lipid can be both anti-inflammatory and pro-inflammatory depending on the location of metabolic activity. Therefore, we aimed to characterize the gene expression of these lipid enzymes and receptors throughout multi-organ diseases via a transcriptomic meta-analysis using the Gene Expression Omnibus (GEO) Database. In our study, we found that distinct AA pathways were expressed in various comorbid conditions, especially those with prominent inflammatory risk factors. Comorbidities, such as hypertension, diabetes, and obesity appeared to contribute to elevated expression of pro-inflammatory lipid mediator genes. Our results demonstrate that expression of inflammatory AA pathway genes may potentiate and attenuate disease; therefore, we suggest further exploration of these pathways as therapeutic targets to improve outcomes.

## 1. Introduction

Inflammation and its resolution play crucial roles in regulating cardiovascular disease in the human body. Metabolic diseases such as obesity, hypertension, and type 2 diabetes are significant risk factors for various pathologies. Inflammatory mediator genes play a role in regulating the vascular endothelium in metabolic diseases across multiple organ systems in the human body. Proposed mechanisms involve the arachidonic acid (AA) pathway which plays a key role in mediating and propagating inflammation in the pulmonary, cardiac, and renal organ systems. AA metabolites, known as eicosanoids, possess variable functions including pro-inflammatory, anti-inflammatory, and pro-resolutory actions [1]. AA metabolic enzymes are expressed ubiquitously in varying degrees across many tissue types, including cardiomyocytes, renal tissue, pulmonary endothelial cells, monocytes, macrophages, and fibroblasts; however, the lipid mediators generated from these enzymatic reactions can be distinctly altered across different tissue and disease states. No study has explicitly examined the shared or divergent AA metabolic perturbations across tissues and comorbid metabolic disease states.

**Figure 1:**
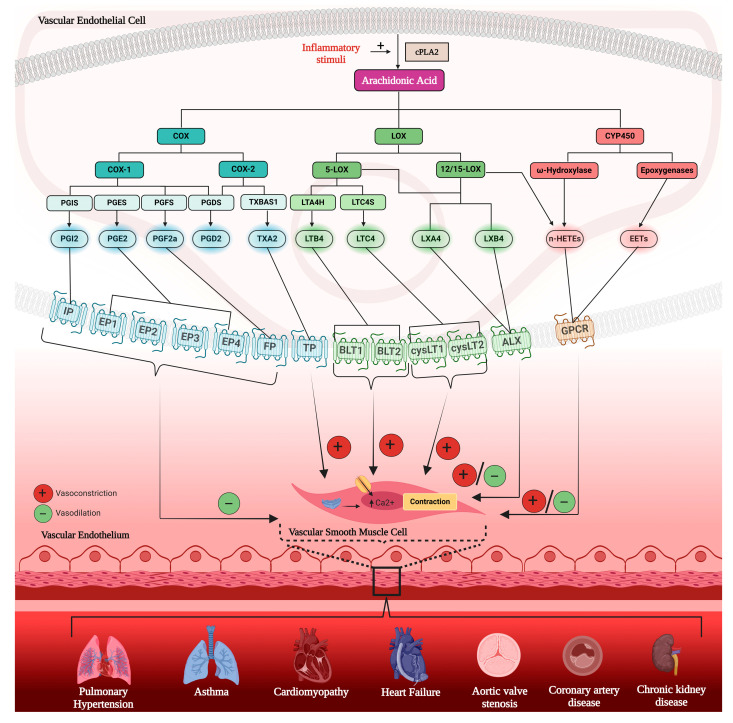
Eicosanoids in human vascular disease. This diagram demonstrates the arachidonic acid (AA) inflammatory pathway involving the primary cyclooxygenase (COX; blue), lipoxygenase (LOX; green), and cytochrome P450 (CYP; red) enzymes to oxidize subsequent metabolites either directly or through other synthases. COX metabolite synthases include prostacyclin synthase (PGI), prostaglandin synthases (PG), and thromboxane synthase (TX) to produce subsequent PGs, PGE2, PGF2a, and PGD2. These metabolites then act on their corresponding receptors, forming complexes leading to intracellular cascades. These complexes include the PGI2-IP, PGE2-EP1-4, and TXA2-TP complexes which vasodilate or vasoconstrict vascular smooth muscle cells. 5-LOX and 12/15-LOX metabolize leukotrienes (LT) and lipoxins (LX). LTs act on BLT1, BLT2, cysLT1, and cysLT2 with a primary vasoconstriction effect, while LXs act on ALX receptor to produce a dual vasodilation and vasoconstriction effect on vascular smooth muscle cells. Finally, CYP450 enzymes include Ѡ-hydroxylase and epoxygenases to produce hydroxyeicosatetraenoic acids (n-HETEs), which have a potent vasoconstriction effect, and epoxyeicosatrienoic acids (EETs), which have a vasodilation effect. These oxidized lipids then contribute to several known human diseases throughout various organ systems, including pulmonary hypertension, asthma, cardiomyopathies, heart failure, aortic valve stenosis, coronary artery disease, and chronic kidney disease.

AA is a free fatty acid generated from the phospholipid membrane of endothelial cells and metabolized by cyclooxygenase (COX)-1 and COX-2 enzymes, expressed by *PTGS1* and *PTGS2* genes (characterized in Table 1), to generate pro-inflammatory thromboxanes (TX) through TXA2 synthase (*TBXAS1* gene) and anti-inflammatory prostaglandins (PG) through PGE and prostacyclin synthase (PGI2; *PTGES* and *PTGIS* genes). AA is also metabolized by 5-lipoxygenase (LOX), 12-LOX, and 15-LOX to generate pro-resolutory lipoxins (LX) and pro-inflammatory leukotrienes (LT) through LTC4 synthase (*LTC4S*) and LTA4 synthase (*LTA4H*). Finally, cytochrome P450 (CYP) enzymes (e.g., *CYP2J2*, *CYP2U1*, *EPHX1*, *EPHX2*) metabolize AA to generate anti-inflammatory epoxyeicosatrienoic acids (EETs) and pro-inflammatory hydroxyeicosatetraenoic acid (HETEs). These generated mediators then act on their subsequent receptors (Figure 1) to induce vasoconstriction or vasodilation of vascular smooth muscle cells and propagate or attenuate an array of diseases in the human body.

Major risk factors for cardiovascular disease in the United States include smoking, diabetes, physical inactivity, body mass index, blood pressure, and increased blood cholesterol levels [2,3]. These risk factors can lead to several comorbid conditions, such as hypertension, diabetes, hyperlipidemia, obesity, and chronic kidney disease (CKD), collectively characterized as metabolic syndrome. Eicosanoid perturbation has been well studied to be associated with metabolic syndrome [1,4,5]. Eicosanoids are extensively involved in cardiovascular disease and endothelial metabolism. Prostanoids notably play a pro- and anti-inflammatory role in the vasculature, increasing vascular permeability and promoting edema, acting synergistically with histamine and bradykinin [6]. There are conflicting data on the role of PGE in vascular diseases. Some studies describe PGE synthase deletion impairing cardiac function and increasing mortality [7]. Others demonstrate increased expression of PGE synthase correlating with increased blood pressure, intima–media thickness, and vascular stiffness [8]. In fact, PGI analogues have been determined to demonstrate anti-proliferative, anti-inflammatory, and endothelial regenerating properties, reduce pulmonary vascular resistance in PH, improve outcomes in CKD, and reduce myocardial remodeling [6,9,10,11]. TXA2 is a potent vasoconstrictor, highly unstable, and rapidly hydrolyzed to the inactive, more stable TXB2. Inhibition of TBX synthase, the enzyme that generates TXA2, has been shown to decrease atherogenesis, decrease blood pressure, and improve vascular dysfunction in hyperlipidemic mice [12,13,14]. *TBXAS1*, the gene coding for TBX synthase has been associated with increased myocardial infarction (MI) risk, changes in platelet function and carotid plaque vulnerability [15,16].

LOX-derived pro-inflammatory LTs and HETEs (also co-derived from CYP hydroxylases) are vasoconstrictors that have been shown to propagate vascular dysfunction. Deletion of 12/15-LOX shows resolution of inflammation post-MI [17]. Conversely, deficiency of *ALOX5*, the gene coding for a key enzyme in LT synthesis, contributed to reduced development of atherosclerosis in metabolic syndrome-modeled mice [18]. *ALOX5* activating protein gene (*ALOX5AP*) has also demonstrated association with increased arterial inflammation, MI, and stroke [17].

Single nucleotide polymorphisms of CYP epoxygenases, which generate EETs, have been found to be associated with atherosclerosis and coronary artery disease (CAD) risk in specific populations [19,20]. EETs, typically vasodilatory and anti-inflammatory, have been found to be cardioprotective. EETs are involved in ameliorating multiple cardiovascular diseases, including CAD, cardiac remodeling, and aortic inflammation [21,22]. Evidence suggests that the actions of EETs are a part of the GPCR signaling pathway, with changes in cAMP signaling; however, it is important to note that it is unclear whether EET-induced changes to cAMP are a result of classic GPCR cellular potentiation [22,23]. Several studies have linked CYP hydroxylase derived 20-HETE with both cardiovascular disorders and renal disease [24]. Therefore, COX, LOX, and CYP enzymes produce metabolites that are pro-inflammatory, anti-inflammatory, and pro-resolutory, with unique functions in different tissues and organ systems.

This study focuses on the cross-comparison of AA metabolism in pulmonary, cardiovascular, and renal pathologies through a tissue and serum transcriptome meta-analysis. We explore metabolic correlations amongst inflammatory comorbidities via investigating specific AA gene expression changes in unique disease states. We also investigate the influence of various metabolic diseases such as obesity, diabetes, and hypertension on the perturbation of these metabolites’ receptor genes across cell types. Table 1 provides a comprehensive summary of genes explored in the present study.

## 2. Materials and Methods

### 2.1. Profile of Datasets

**Figure 2:**
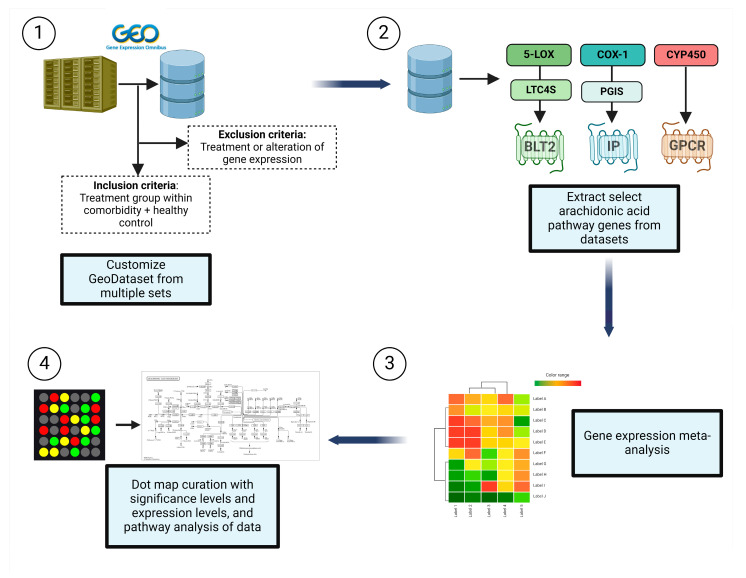
Methodology of data curation and analysis. (**1**) Datasets containing genes of interest were retrieved from the Gene Expression Omnibus (GEO) Database. Inclusion criteria included groups within the comorbidity of interest and a healthy control. (**2**) We extracted the select arachidonic acid (AA) pathway genes from AA pathways including the lipoxygenase (LOX), cyclooxygenase (COX), and CYP450 (CYP) enzymes. (**3**) Gene expression values were collated from these datasets and (**4**) plotted on a dot plot to simultaneously display differential expression levels as logarithm to base 2 of the fold change (log2FC) and significance compared with control (healthy patients from the same GEO dataset). In addition, pathway analyses were conducted to determine predominant pathways in comorbid groups.

In the current study (Figure 2), we examined the expression of AA cascade enzyme- and receptor-related genes in target organ systems including pulmonary, renal, cardiac, and peripheral blood mononuclear cells (PBMCs) from 1631 patients across a variety of common comorbidities including hypertension, diabetes, CKD, pulmonary hypertension (PH), asthma, cardiomyopathies (CM), heart failure (HF), aortic stenosis (AS), CAD, and obesity, as shown in Table 2.

### 2.2. Data Collection

Differential gene expressions were curated from data deposited in the Gene Expression Omnibus (GEO) datasets and the European Molecular Biology Laboratory (EMBL). Intensive queries in these databases in the form of “[tissue] and [hypertension, diabetes, obesity, CM, HF, AS, and asthma]” were performed using the NCBI and iLINCS (ilincs.org) websites. All hits were considered. Excluded data comprised DataSeries not containing disease states, lacking healthy controls for the tissues concerned, or involving treatments and/or gene alteration of any form.

### 2.3. Data Analysis

**Figure 3:**
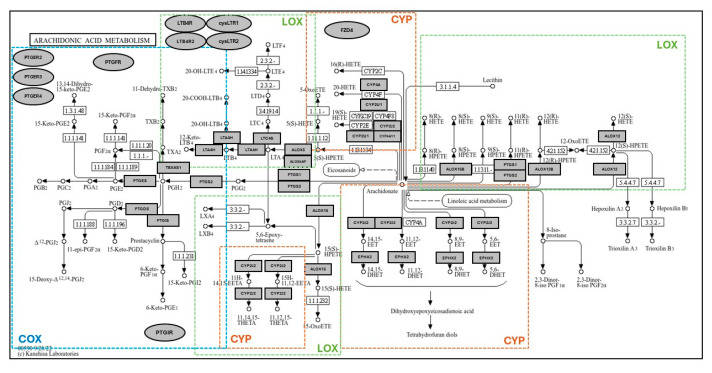
KEGG pathway map of arachidonic acid (AA) metabolism with boxed areas displaying genes for which expression has been quantified in this study. In each figure, differentially expressed AA metabolism enzyme-related genes are mapped to the KEGG pathway map in individual studies. Outlines demonstrate the area of cyclooxygenase (COX), lipoxygenase (LOX), and cytochrome P450 (CYP) metabolism, and their areas of overlap. Receptor-related genes are depicted on the periphery of the map as oval shapes. Expression levels are demonstrated as red (significantly higher expression), blue (significantly lower expression), or gray (non-significant change in expression), with significance expressed as *p* < 0.05.

**Figure 4:**
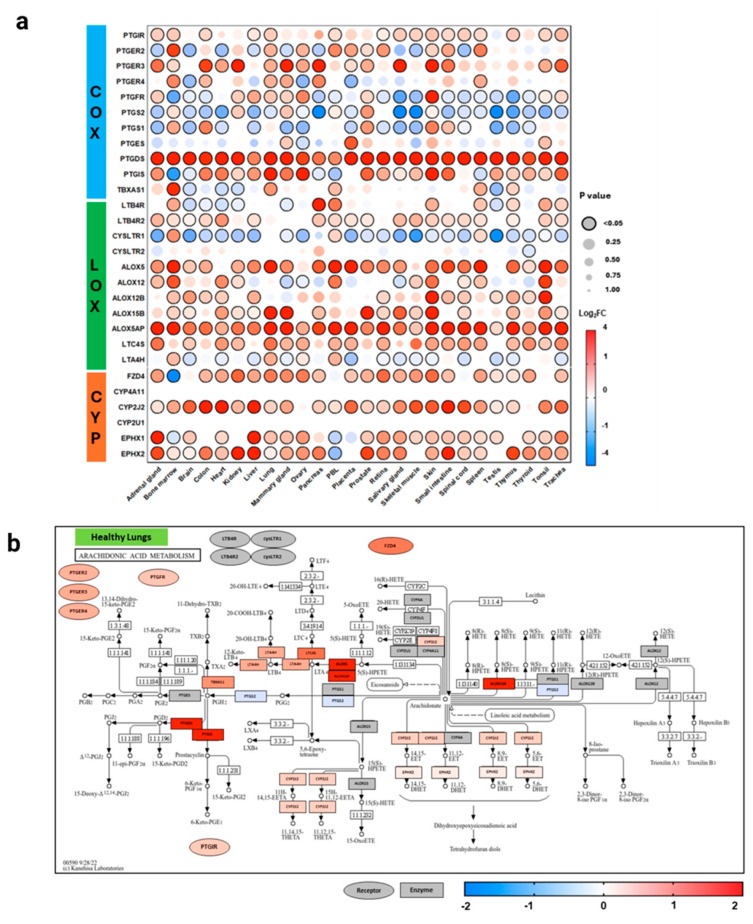

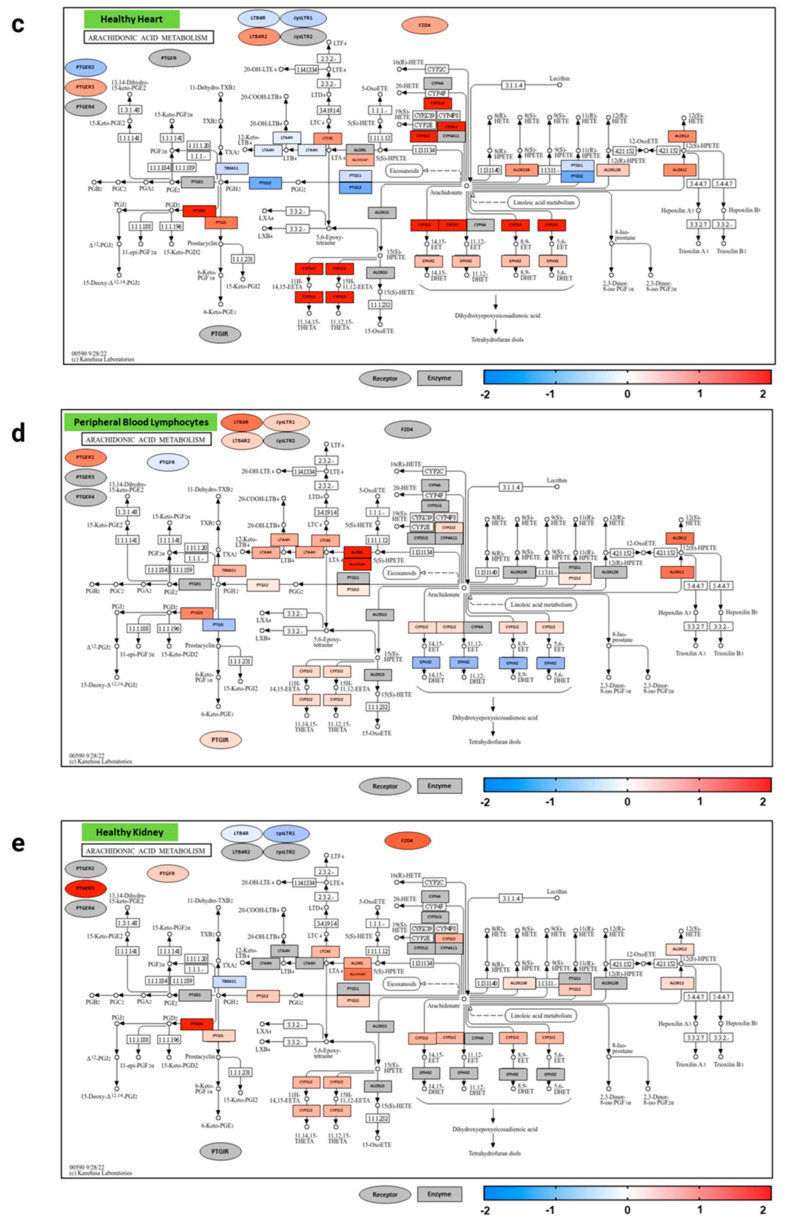
Arachidonic acid pathway gene expression across healthy human tissues. This graph (**a**) demonstrates the expression of select arachidonic acid (AA) pathway genes in the lipoxygenase (LOX), cyclooxygenase (COX), and cytochromeP450 (CYP) enzymatic oxidation pathways in healthy volunteers across 26 different body sites. Expression is shown as logarithm to base 2 of the fold change (log2FC) relative to Universal Human Reference RNA, derived from the GEO dataset (GDS3113; *n* = 3). Heatmap coloration is set in a scale from −4 (blue) to +4 (red), and values beyond this range are shown as −4 or +4, respectively. Statistical significance is displayed proportional to dot size, where the largest dot size has the highest statistical significance and a threshold of *p* < 0.05 is marked by a black border. KEGG pathway enrichment analysis graphs of AA metabolism isolate healthy lung (**b**), heart (**c**), kidney (**d**), and peripheral lymphocyte (**e**) data and demonstrate a functional pathway schematic, with genes colored according to the expression levels shown in (**a**). Rectangles demonstrate enzyme-related gene expression and ovals show receptor-related gene expression, located in the periphery of the KEGG map. Additional information for each dataset can be found in Appendix A. COX = cyclooxygenase, LOX = lipoxygenase, CYP = cytochrome P450.

Based on the methodology our group has previously demonstrated [25], differential expression analysis and statistical analysis was performed using the GEO2R (NCBI) interactive web tool, and the iLNCS interactive web platform for the analysis of the Library of Integrated Network-Based Cellular Signatures (LINCS) [26,27]. Expression values in healthy tissues are reported as logarithm to base 2 of the fold change (log2FC) relative to Universal Human Reference RNA, quantified in the GEO dataset GDS3113. For all comorbidity disease condition data, expression levels are described in log2FC in relation to each unaffected control group specific to each DataSeries. Dot plots were generated using R programming language and ggplot2 with heatmap style coloration indicating log2FC [28]. Dot sizes are proportional to the *p*-value where largest dot sizes indicate highest statistical significance and black border indicates a *p*-value < 0.05. All log2FC values outside of the −2 to +2 range are shown as either −2 or +2. KEGG (Kyoto Encyclopedia of Genes and Genomes) [29] pathway maps were generated from disease states with notable and significant results, displayed in heatmap style coloration matched with the dot plots. Differentially expressed genes were mapped to the KEGG pathway map by hand. Receptor-related genes are expressed on the periphery of the map as oval shapes and enzyme-related genes as rectangles. Expression levels are demonstrated as red (significantly higher expression), blue (significantly lower expression), or gray (non-significant change in expression), with adjusted *p* < 0.05 assigned as being significantly and differentially expressed. A modified KEGG map of AA metabolism is shown in Figure 3. The healthy dataset (Figure 4) demonstrates log2FC values from −4 to +4 range, with values outside the range shown as −4 to +4. DataSeries in this analysis are described in Appendix A. 

## 3. Results

### 3.1. Distribution of Expression in Healthy Tissues

To evaluate the role of AA pathway genes and their expression in pulmonary, cardiovascular, and renal diseases, expression data were initially analyzed from healthy tissue across 26 different body sites from three individuals (Figure 4). In healthy human tissues, there was a diverse expression of the COX (*PTGFR*, *PTGIR*, *PTGER2*, *PTGER3*, *PTGER4*, *PTGS2*, *PTGS1*, *PTGES*, *PTGIS*, *TBXAS1*), LOX (*LTB4R*, *LTB4R2*, *CYSLTR1*, *CYSLTR2*, *ALOX5*, *ALOX12*, *ALOX15*, *LTC4S*), and CYP (*FZD4*, *EPHX1*, *EPHX2*, *CYP2U1*, *CYP2J2*, *CYP4A11*) pathway genes. COX genes, mainly producing anti-inflammatory and pro-inflammatory metabolites, were downregulated more than LOX or CYP genes, across organ systems. These results from our analysis affirm previous known roles of AA metabolites in physiological states [30].

### 3.2. Expression in Human Pulmonary Disease Comorbid States

**Figure 5:**
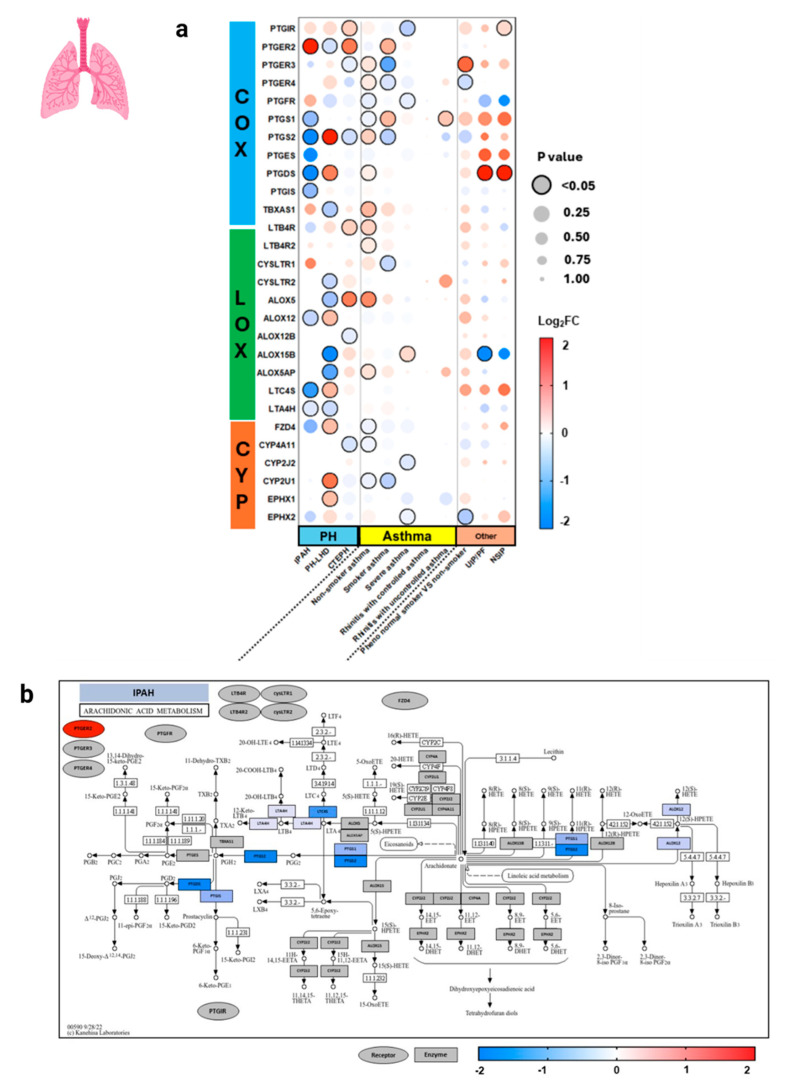

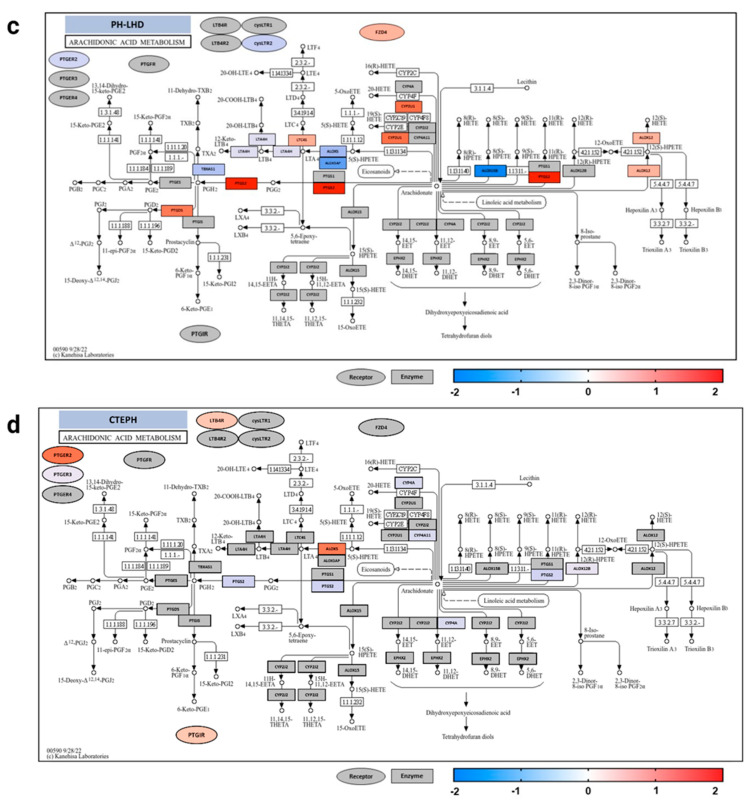
Arachidonic acid eicosanoid pathway gene expression in human pulmonary diseases. The World Health Organization (WHO) defines 5 groups of pulmonary hypertensions (PH), in which here (**a**) we have specifically studied group 1 isolated pulmonary arterial hypertension (IPAH) (**b**), group 2 PH due to left-heart disease (PH-LHD) (**c**), and group 4 chronic thromboembolic PH (CTEPH) (**d**). Expression is shown as logarithm to base 2 of the fold change (log2FC) relative to Universal Human Reference RNA, derived from 10 datasets. Heatmap coloration (**a**) is set in a scale from −2 (blue) to +2 (red), and values beyond this range are shown as −2 or +2, respectively. Statistical significance is displayed proportional to dot size, where the largest dot size has the highest statistical significance and a threshold of *p* < 0.05 is marked by a black border. Pathway enrichment analysis is conducted (**b**–**d**) using the heatmap coloration (−2 to +2) on various genes placed on a KEGG pathway map of AA metabolism. Rectangles demonstrate enzyme-related gene expression and ovals show receptor-related gene expression, located in the periphery of the KEGG map. Additional information for each dataset can be found in Appendix A. COX = cyclooxygenase, LOX = lipoxygenase, CYP = cytochrome P450, IPAH = isolated pulmonary arterial hypertension, PH-LHD = pulmonary hypertension due to left-heart disease, CTEPH = chronic thromboembolic pulmonary hypertension, UIP = usual interstitial pneumonia, PF = pulmonary fibrosis, NSIP = nonspecific interstitial pneumonia.

In pulmonary tissues (Figure 5), World Health Organization (WHO) group I idiopathic pulmonary arterial hypertension (IPAH) and group II PH due to left-heart disease (PH-LHD) demonstrated distinctly different patterns of AA metabolism. LOX and CYP pathway genes were significantly upregulated in PH-LHD, while IPAH demonstrated a greater significant downregulation in COX genes, as demonstrated in the pathway analysis. Interestingly, *PTGER2*, *PTGS2*, *PTGDS*, *ALOX12*, and *LTC4S* were inversely expressed in IPAH and PH-LHD, suggesting that divergent mechanisms are at play. Group 4 PH, chronic thromboembolic PH (CTEPH), also demonstrated inverse expression of *PTGER2* and *PTGS2* in a similar expression pattern to IPAH. Non-smoker asthma and smoker asthma demonstrated mild significant changes in diverse AA genes, while the other pulmonary disease states did not show many significant changes.

### 3.3. Expression in Cardiac Tissues and Comorbid States

**Figure 6:**
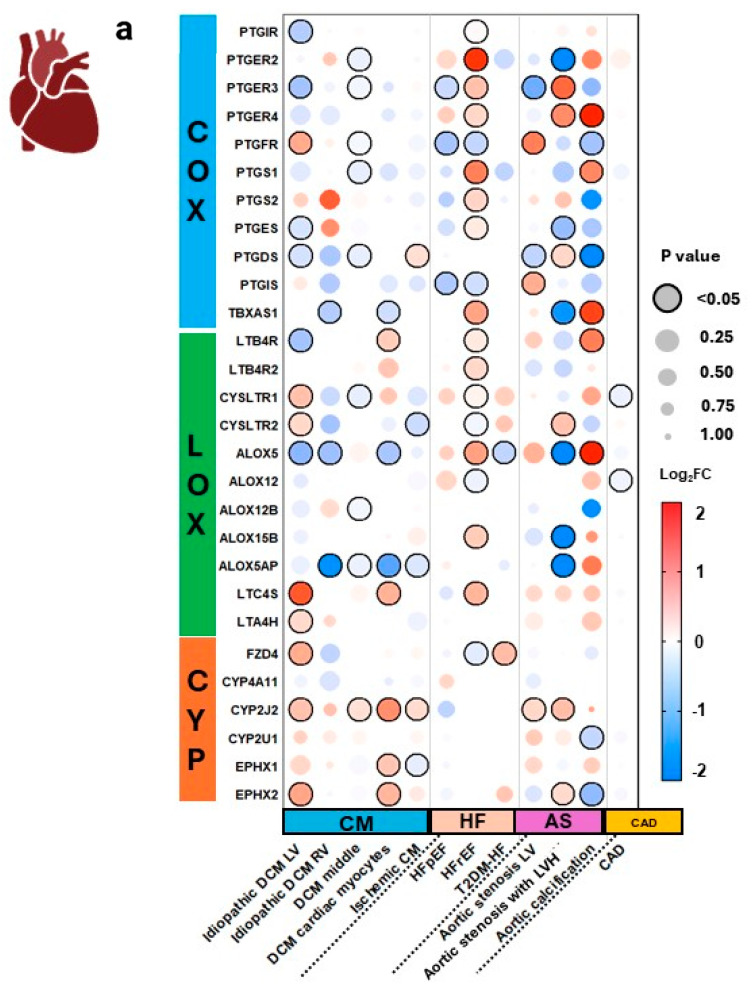

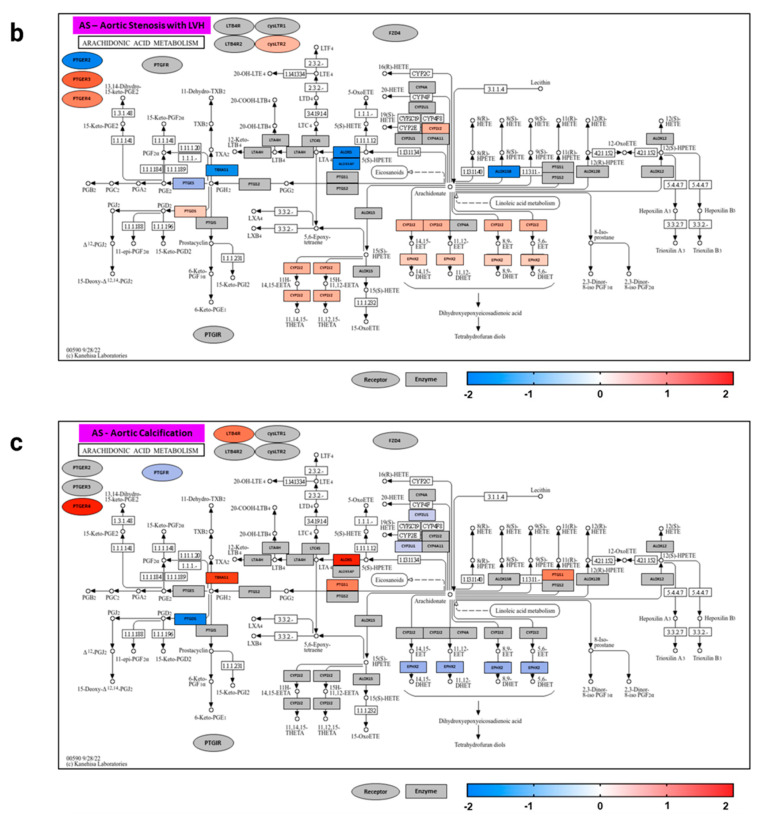
Arachidonic acid eicosanoid pathway gene expression in human cardiovascular diseases. Expression of select arachidonic acid (AA) pathway genes across 12 datasets representing various cardiovascular comorbidities (**a**). Pathway analysis of aortic stenosis (AS) with left ventricular hypertrophy (LVH) and with calcification are mapped according to expression (**b**,**c**). Expression is shown as logarithm to base 2 of the fold change (log2FC) relative to Universal Human Reference RNA, derived from 10 datasets. Heatmap coloration is set in a scale from −2 (blue) to +2 (red), and values beyond this range are shown as −2 or +2, respectively. Statistical significance is displayed proportional to dot size, where the largest dot size has the highest statistical significance and a threshold of *p* < 0.05 is marked by a black border. Pathway enrichment analysis is conducted using the heatmap coloration (−2 to +2) on various genes placed on a KEGG pathway map of AA metabolism (**b**,**c**). Rectangles demonstrate enzyme-related gene expression and ovals show receptor-related gene expression, located in the periphery of the KEGG map. Additional information for each dataset can be found in Appendix A. COX = cyclooxygenase, LOX = lipoxygenase, CYP = cytochrome P450, CM = cardiomyopathy, DCM = dilated cardiomyopathy, LV = left ventricle, RV = right ventricle, HF = heart failure, HFpEF = heart failure with preserved ejection fraction, HFrEF = heart failure with reduced ejection fraction, T2DM-HF = heart failure due to diabetic cardiomyopathy, AS = aortic stenosis, LVH = left ventricle hypertrophy, CAD = coronary artery disease.

In cardiac tissues (Figure 6), we observed that CM tissue from the left ventricle (LV), right ventricle (RV), middle, and cardiac myocytes demonstrated a similar pattern in expression, with the LV demonstrating the greatest downregulation of COX and LOX genes, and upregulation of LOX and CYP genes.

In the HF groups, heart failure with reserved ejection fraction (HFrEF) demonstrates greater downregulation in CYP enzyme-related genes (CYP2A6, CYP2D6) and FZD4 receptor-related gene; LOX enzyme-related genes (ALOX12, ALOX15B) and cysLTR2 receptor-related genes (CYSLTR1, CYSLTR2); and COX genes, including COX receptor-related genes (PTGFR, PTGIR, PTGIS). These changes also coincide with heart failure with preserved ejection fraction (HFpEF), which demonstrates downregulation of PTGR, PTGIS, and PTGER3 genes.

AS groups show significant variability, with aortic calcification demonstrating upregulation of LOX enzyme ALOX5 and pro-inflammatory LOX-derived LT receptor-related gene LTB4R, suggesting increased LT production. Upregulation of COX pathway gene TBXAS1, which produces pro-inflammatory TXs, also contributes to a pro-inflammatory state. Additionally, AS from LVH demonstrates upregulated anti-inflammatory COX receptor-related genes PTGER2 and PTGER4, and LOX-derived LT receptor-related gene CYSLTR2, but downregulated TBXAS1 and ALOX5, suggesting different mechanisms causing aortic valve disease and aortic calcification. These data are interesting, as valve disease and calcification typically occur in a continuum. The expression levels demonstrate that pro-inflammatory lipid mediators may be enhancing calcification.

### 3.4. Expression in Renal Diseases and Comorbid States

**Figure 7:**
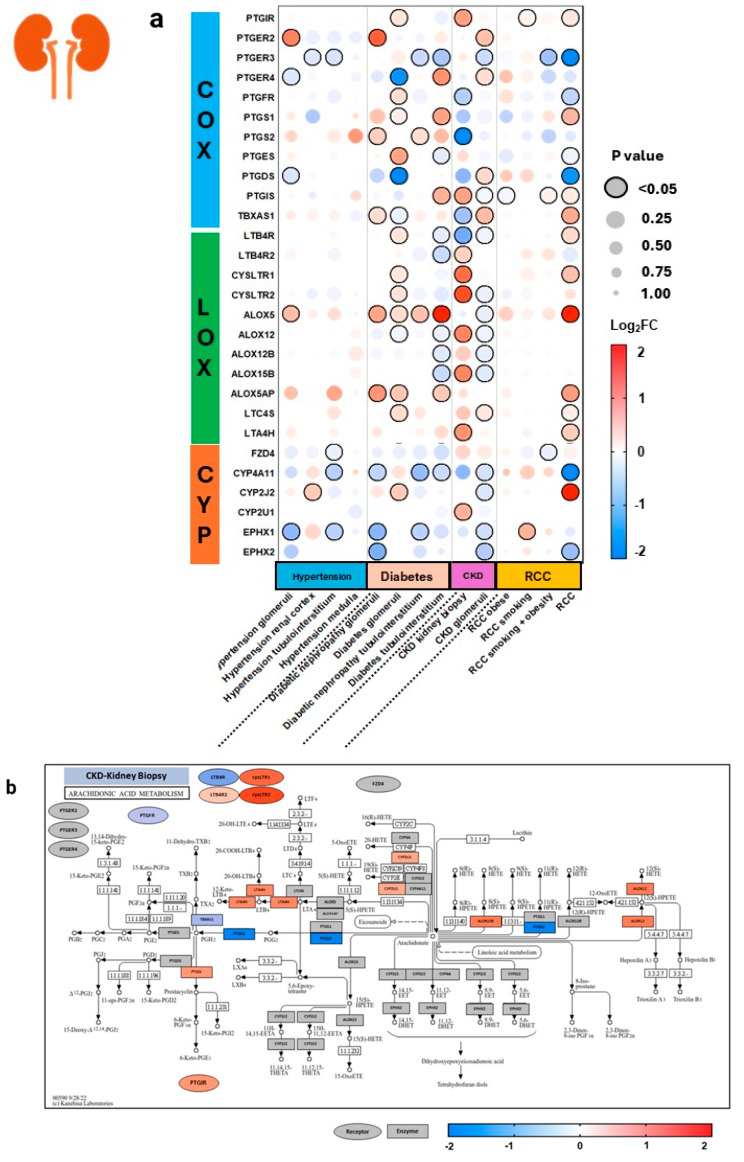

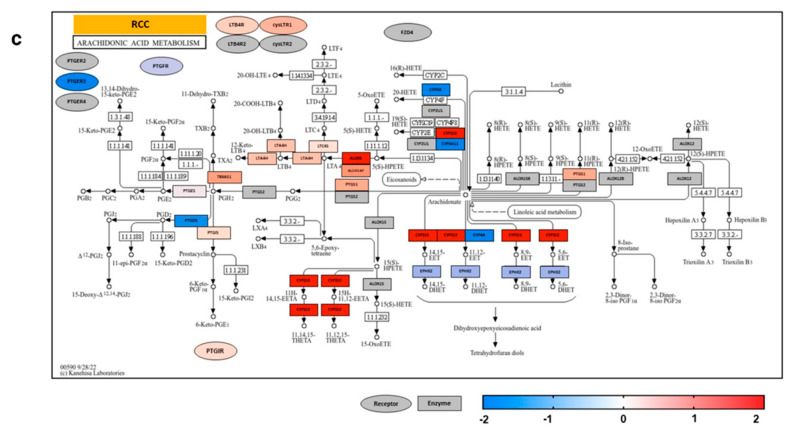
Arachidonic acid eicosanoid pathway gene expression in human renal diseases. Expression of select arachidonic acid (AA) pathway genes across 14 datasets representing various renal comorbidities (**a**). Pathway analyses are mapped for chronic kidney disease (CKD) and renal cell carcinoma (RCC) (**b**,**c**). Expression is shown as logarithm to base 2 of the fold change (log2FC) relative to Universal Human Reference RNA, derived from 10 datasets. Heatmap coloration is set in a scale from −2 (blue) to +2 (red), and values beyond this range are shown as −2 or +2, respectively. Statistical significance is displayed proportional to dot size, where the largest dot size has the highest statistical significance and a threshold of *p* < 0.05 is marked by a black border. Pathway enrichment analysis is conducted using the heatmap coloration (−2 to +2) on various genes placed on a KEGG pathway map of AA metabolism (**b**,**c**). Rectangles demonstrate enzyme-related gene expression and ovals show receptor-related gene expression, located in the periphery of the KEGG map. Additional information for each dataset can be found in Appendix A.COX = cyclooxygenase, LOX = lipoxygenase, CYP = cytochrome P450, CKD = chronic kidney disease, RCC = renal cell carcinoma.

Within the diabetes group from renal tissue (Figure 7), there was quite an array of changes in expression, with renal glomeruli demonstrating upregulation of various COX, LOX, and CYP pathway genes. Interestingly, the tubulointerstitium of diabetic patients showed downregulation of anti-inflammatory CYP enzyme-related genes *CYP4A11*, *EPHX1*, and *EPHX2*. In diabetes, the tubulointerstitium and glomeruli of kidney tissue demonstrated upregulation of *ALOX5* and *ALOX5AP*, and mostly downregulation of COX genes. Kidney biopsy from CKD patients shows upregulation of LOX-5 derived pro-inflammatory LT receptor-related genes *cysLTR1*, *cysLTR2*, and *LTB4R2*, as well as upregulation of LOX enzyme-related genes *ALOX15B* and *ALOX12*. In the renal cell carcinoma (RCC) group, the greatest increase is seen in RCC without comorbid obesity or smoking, as seen with upregulation of *PTGS1*, which aligns with current literature studying that COX-1 expression correlates with clinicopathological features of RCC. However, no significant changes were seen in COX-2 (*PTGS2* gene) expression, despite COX-2’s well-established role in the inflammation-carcinoma sequence in various epithelial cancers [31]. RCC also demonstrates upregulation of *ALOX5*, *TBXAS1*, and *cysLTR1*, supporting literature on the role of TBXA2 in upregulating renal cancer and 5-LOX in progressing carcinogenesis [32,33].

### 3.5. Expression in Blood Tissues in Comorbid States

**Figure 8:**
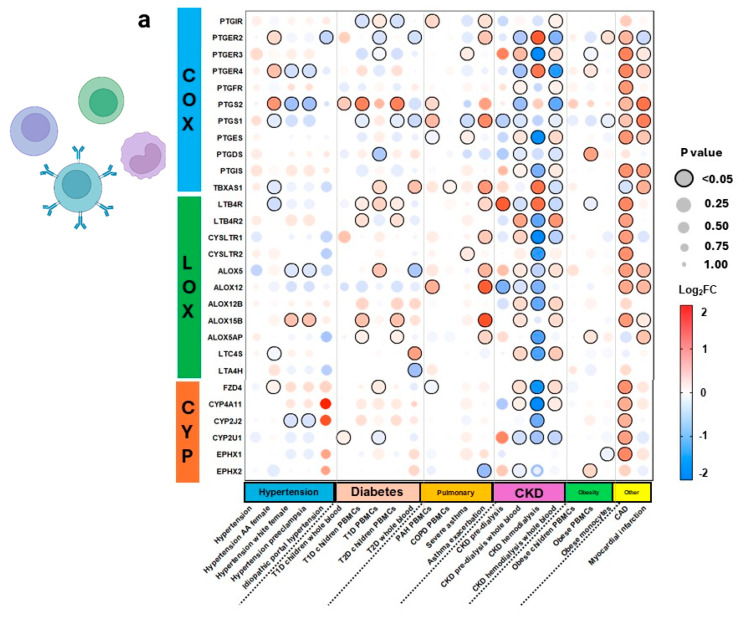

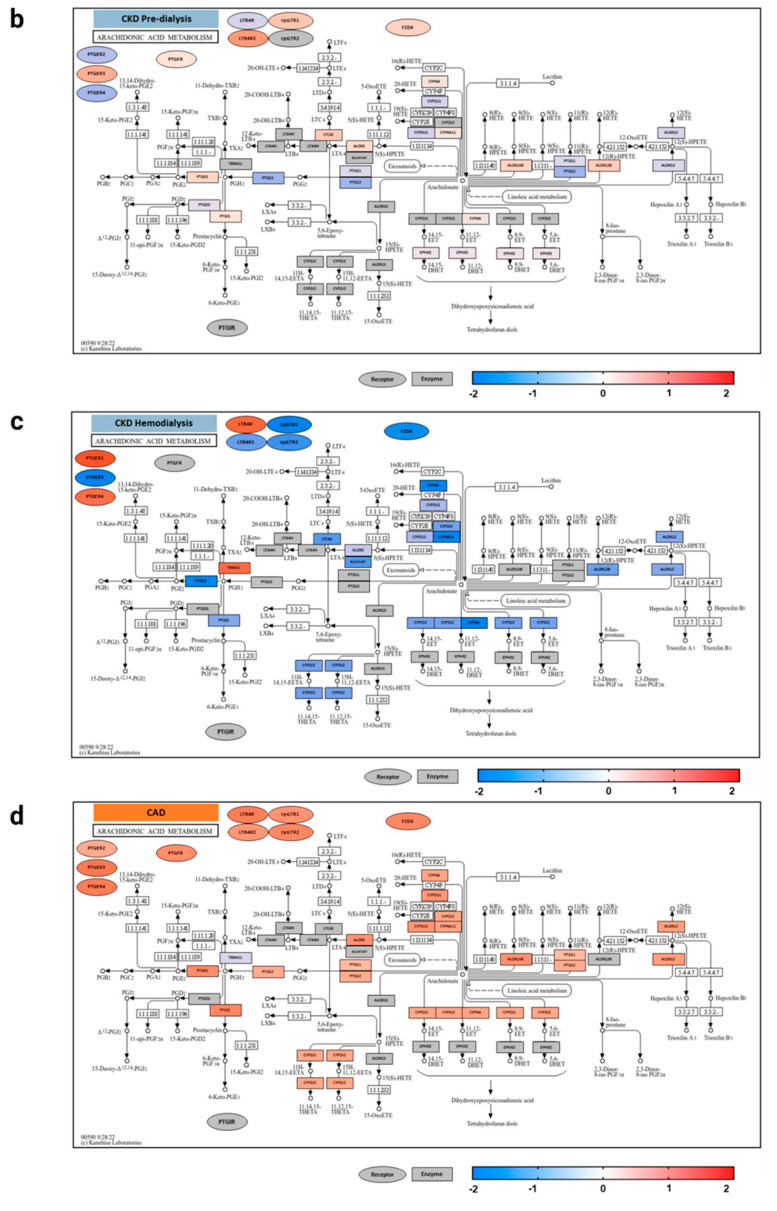
Arachidonic acid eicosanoid pathway gene expression in blood tissues across comorbidities. Expression of select arachidonic acid (AA) pathway genes across 23 datasets representing gene expression in peripheral blood monocyte cells (PBMCs) (**a**). Pathway analysis is shown for disease states with significant expression, including chronic kidney disease (CKD) pre-dialysis, after hemodialysis, and coronary artery disease (CAD) (**b**–**d**). Expression is shown as logarithm to base 2 of the fold change (log2FC) relative to Universal Human Reference RNA, derived from 10 datasets. Heatmap coloration is set in a scale from −2 (blue) to +2 (red), and values beyond this range are shown as −2 or +2, respectively. Statistical significance is displayed proportional to dot size, where the largest dot size has the highest statistical significance and a threshold of *p* < 0.05 is marked by a black border. Pathway enrichment analysis is conducted using the heatmap coloration (−2 to +2) on various genes placed on a KEGG pathway map of AA metabolism (**b**–**d**). Rectangles demonstrate enzyme-related gene expression and ovals show receptor-related gene expression, located in the periphery of the KEGG map. COX = cyclooxygenase, LOX = lipoxygenase, CYP = cytochrome P450, AA = arachidonic acid, T1D = type 1 diabetes, T2D = type 2 diabetes, PBMCs = peripheral blood mononuclear cells, PAH = pulmonary arterial hypertension, COPD = chronic obstructive pulmonary disease, CKD = chronic kidney disease, CAD = coronary artery disease.

AA pathway genes were expressed more in CKD comorbidities and CAD compared to other conditions in PBMCs and blood samples (Figure 8). Specifically, CKD pre-dialysis demonstrated increased AA enzymes and receptors across all pathways. In contrast, CKD hemodialysis significantly downregulated most AA pathway genes, except for COX receptor-related genes *PTGER2* and *PTGER4*, enzyme-related gene *TBXAS1*, and CYP receptor-related gene *FZD4*. The downregulation of genes from pre-dialysis to hemodialysis in CKD suggests contribution of AA metabolites in CKD inflammation. Additionally, CAD demonstrated remarkable increases in almost all select AA genes.

## 4. Discussion

AA and its various pathways of metabolism have been well described in literature for their role in inflammation in the human body [30]. AA metabolism varies across different cell types and organs, with eicosanoid levels continuously fluctuating. Indeed, studies have shown that when COX-2 and 5-LOX gene expression is inhibited, there is compensatory upregulation of COX-1, demonstrating that AA metabolism has the potential to “class-switch” to induce expression of other enzymes [34,35]. In addition, PGE2, which has been long thought to be pro-inflammatory, was later discovered to also have anti-inflammatory properties in several organ systems [36,37]. The diverse role of aspirin (a COX inhibitor) in treating various conditions, from HF to cancer, emphasizes the importance of eicosanoids throughout various inflammatory vascular conditions and highlights the need for further inspection into the pathological expression of eicosanoid genes [38,39,40]. In chronic inflammatory and autoimmune diseases, such as rheumatoid arthritis and systemic lupus erythematosus, the upregulation of AA pathway genes is well studied [41]. However, tissue-specific regulatory mechanisms of AA metabolism in autoimmune vs. non-autoimmune diseases entails further study. Despite aspirin’s unique role in COX and prostanoid signaling, studies have demonstrated that long-term use of aspirin in a 20-year cohort was associated with slight to moderately reduced risk of several cancers (e.g., colon, rectal, esophageal, gastric, leukemia, etc.), but not cancer overall, and even increased risk for some cancers (e.g., lung and bladder) [42]. This variability reiterates the complex role of eicosanoids in different organ systems, and the critical need to develop an understanding of eicosanoid expression. It is essential to investigate eicosanoids beyond the COX pathway that regulate inflammation, to potentially discover new therapeutic targets that may augment or supplement anti-inflammatory properties of various drugs such as aspirin.

In the present study, we discovered that in healthy tissue across 26 different organs, there was downregulation of genes associated with COX and LOX metabolites, and upregulation of CYP enzyme- and receptor-related genes. We also observed uncoupling of COX-1 and COX-2 enzymes with downstream synthase enzymes, such as *PTGDS*, *PTGES*, and *PTGIS*. This is converse to Kihara et al.’s quantitative model of the eicosanoid metabolic pathway, which integrated both lipidomics and transcriptomics in primary macrophages to understand the complex characteristics of eicosanoid networks [43]. This study demonstrated downstream coupling between COX and terminal enzymes, such as PGD2-synthase and TXA2-synthase [43]. LOX enzymes *ALOX5* and *ALOX5AP* demonstrated greater coupling with downstream *LTC4S* gene.

Eicosanoids help manage systemic inflammation and play a key role in lung health [44]. An imbalance of these metabolites can lead to conditions like PH, asthma, and other lung diseases. PGs and TXs, specifically 11β-dhk-PGF2α, are linked to lung blood vessel constriction and increased airway resistance [45]. In the present study, increased expression of diverse AA pathway genes in WHO group 1 PAH, group 2 PH-LHD, and group 4 CTEPH signifies altered disease mechanisms. This aligns with the distinguished pathophysiology of each PH group. For instance, PH-LHD involves a gradual increase in passive left-sided pressures which contribute to subsequent vascular remodeling by various inflammatory biomarkers and right heart dysfunction [46]. PAH, on the other hand, is an inflammatory condition resulting from vascular remodeling directly due to pulmonary disease. Interestingly, CTEPH follows a closer AA expression pattern with Group I PAH, suggesting divergent inflammatory mediators compared to Group II PH-LHD, which may have greater systemic inflammatory involvement. The distribution of upregulated and downregulated AA genes in these PH comorbidities highlights the role of AA eicosanoids in vascular remodeling across these conditions.

In cardiovascular comorbid states, an increase in AA genes in DCM LV was observed compared to other portions of the heart and ischemic DCM. This suggests the unique role of AA inflammatory mediators in LV remodeling that potentiate disease. Indeed, Chauhan et al. demonstrated that AA genes are significantly perturbed across cardiomyopathies, with potential influence on fibrosis during cardiomyopathy [47]. HFrEF tissue demonstrated greater expression of AA pathway genes, yet little expression in HFpEF. Metabolic syndrome, a potent risk factor of HFpEF, has been studied to induce myocardial dysfunction and remodeling [46,48,49]. Previous studies demonstrated the upregulation of PGs and oxidized lipids in HFpEF, suggesting that pro-inflammatory eicosanoid mediators contribute to the pathogenesis of HFpEF [49]. In the present study, the mild gene expression in transcriptome analysis of HFpEF cardiac tissue contrasts with recent studies showing increased circulating AA lipid metabolites, which potentiate HFpEF. These findings may suggest the role of systemic inflammation in modulating AA lipids beyond myocardial tissue metabolism [49]. HFrEF, on the other hand, depicts a myocardial tissue-localized mechanism. 

Another condition associated with metabolic syndrome is aortic calcification [50]. Nephrectomy animal models that demonstrated CKD-induced vascular calcification showed increased ALOX15 and 11-HETE, 12-HETE, and 15-HETE in calcified aortas [51]. Saito et al. has also previously demonstrated that n-3 (omega-3) eicosapentanoic acid (EPA), another polyunsaturated fatty acid derivative, significantly suppressed arterial and aortic calcification in vitro and in vivo [52]. EPA inhibits osteoblastic change and mineralization in vascular cells, affecting both aortic calcification and CKD [52,53]. N-3 PUFAs act as potent resolvers of inflammation, essentially balancing the pro-inflammatory, vasoconstricting, thrombus-forming role of n-6 PUFAs like AA. Similarly, in aortic valve stenosis, valvular COX prostanoids, PGE2 and PGF2a, both mediators of calcium resorption and bone remodeling, have been shown to be elevated [54]. Furthermore, Van Driel et al. demonstrated increased eicosanoids in the profile of AS patients, suggesting increased inflammatory activity [55].

In the present study, we also demonstrate extensive AA pathway gene expression in CKD. Interestingly, when investigating the transcriptome of blood samples, we noticed that AA gene expression pre-dialysis to hemodialysis changed dramatically, with upregulation of COX, LOX, and CYP genes pre-dialysis to downregulation during hemodialysis. CYP enzymes were slightly upregulated in whole blood of hemodialysis patients. In contrast, Gollasch et al. studied the role of hemodialysis on plasma oxidized lipids to find that CYP metabolites were increased after dialysis, with no change in LOX enzymes [56]. Surapaneni et al. demonstrated higher levels of n-3 (omega-3) PUFAs, which have a protective effect against end-stage kidney disease, and higher levels of AA having a positive association with risk of end-stage kidney disease [57]. CYP hydroxylase generated 20-HETE has also been studied in association with hypertension and CKD. The interaction of 20-HETE with GPCR75 in endothelial cells initiates intracellular signaling pathways like MAPK and Rho kinases, which contribute to endothelial dysfunction [58,59]. This is characterized by a reduction in nitric oxide levels and an increase in reactive oxygen species. In vascular smooth muscle cells, 20-HETE leads to a decrease in membrane potential, which causes a vasoconstrictive effect. Conversely, 20-HETE serves a dual role by acting as a natriuretic factor in the proximal tubule of kidney cells, inducing a renoprotective mechanism [60]. Indeed, urinary 20-HETE is being recognized as a promising non-invasive biomarker for diabetic kidney disease [58,61,62].

### Limitations

There are a few limitations to this analysis. First, as gene expression data were collected from numerous studies over two databases, there is an inherent lack of uniformity in methodology. Details of each selected study can be found in Appendix A. Additionally, tissue types only represent the genetic status at one point in time, and do not characterize the entire manifestation of disease state. Our study is also limited by lack of downstream enzymatic protein expression. Other limitations to data curation include different sample sizes and lack of equivalent number of healthy controls. This heterogeneity should be considered when determining the strength of associations found between gene expression and certain diseases.

Another limitation to our study is that we focused our research on select AA pathway genes that were associated with lipid mediators well-characterized in literature. This limitation in scope, while necessary for readability, does not fully encompass the vast nature of polyunsaturated fatty acid metabolism, which consists of many other lipids that carry potent properties in inflammation and disease. Two methods to analyze the AA fatty acid lipidome are via transcriptomic analysis, as demonstrated in the present study, and lipidomic analysis. Lipidomic analysis focuses on quantifying levels of downstream lipid mediators to understand their function. As true lipid expression is influenced by epigenetics, post-transcriptional, post-translational, and enzyme activity, the transcriptome may not be representative of lipid levels; however, circulating eicosanoids are rapidly cleared by the lungs and kidneys and seldom exist in a stable form. While mass spectrometry-based methods for lipidomics may be the only feasible method that can sensitively and specifically detect stable, circulating eicosanoids, there is still a paucity of these types of studies [63,64]. Therefore, in the present state-of-the-art, gene expression allows us to determine associations between known genes and disease states and characterize involved pathways against comorbidities to note any differences in regulating mechanisms, which may be followed-up by more specific measurements. Furthermore, understanding the gene expression of receptors for these metabolites is necessary, because circulating lipids need to bind and interact with these receptors to exert their effects.

## 5. Conclusions

Our results support the unique, specific roles eicosanoids maintain in different tissue types. We provide a broad, cross-comparative display of AA pathway gene perturbation across major organ systems and vascular inflammatory comorbidities. This study highlights the novel and clinically relevant aspects of AA metabolism throughout pulmonary, cardiovascular, and renal tissue to generate a functional understanding of gene expression and enzyme–receptor coupling. We believe this information represents an underappreciated aspect of human physiology and systems which will further benefit future efforts in personalized medicine.

## Figures and Tables

**Table 1:** Arachidonic pathway gene summary.

Pathway	Protein (Enzyme/Receptor)	Protein Abbreviation	Gene Symbol
**COX**	***COX enzymes***		
	Cyclooxygenase-1	COX-1	*PTGS1*
	Cyclooxygenase-2	COX-2	*PTGS2*
	Prostaglandin synthase	PGE synthase	*PTGES*
	Prostacyclin synthase	PGI synthase	*PTGIS*
	Thromboxane synthase	TXA2 synthase	*TBXAS1*
	***COX receptors***		
	Prostaglandin F receptor	FP	*PTGFR*
	Prostaglandin receptor(s)	EP2	*PTGER2*
	Prostaglandin receptor(s)	EP3	*PTGER3*
	Prostaglandin receptor(s)	EP4	*PTGER4*
	Prostacyclin receptor	IP	*PTGIR*
**LOX**	***LOX enzymes***		
	Lipoxygenase-5	LOX-5	*ALOX5*
	Lipoxygenase-5 activating protein	LOX-5AP	*ALOX5AP*
	Lipoxygenase-12	LOX-12	*ALOX12*
	Lipoxgyenase-12b	LOX-12b	*ALOX12B*
	Lipoxgyenase-15b	LOX-15b	*ALOX15B*
	Leukotriene synthase(s)	LT synthase	*LTC4S*
	Leukotriene synthase(s)	LT synthase	*LTA4H*
	***LOX receptors***		
	Leukotriene receptor(s)	LT receptor	*BLT1*
	Leukotriene receptor(s)	LT receptor	*BLT2*
	Cysteinyl-Leukotriene receptor(s)	cysLT receptor	*cysLTR1*
	Cysteinyl-Leukotriene receptor(s)	cysLT receptor	*cysLTR2*
**CYP**	***CYP enzymes***		
	CytochromeP450 2J2	CYP2J2	*CYP2J2*
	CytochromeP450 2U1	CYP2U1	*CYP2U1*
	CytochromeP450 4A11	CYP4A11	*CYP4A11*
	Epoxide Hydrolase 1	EPHX1	*EPHX1*
	Epoxide Hydrolase 2	EPHX2	*EPHX2*
	***CYP receptor***		
	G-protein coupled receptor	GPCR	*FZD4*

**Table 2:** Comorbidities sample size.

Disease	*n*	Control	*n*
Hypertension	94	Normotensive	61
Diabetes	112	Normoglycemic	101
Chronic kidney disease	157	No renal disease	68
Pulmonary hypertension	30	No pulmonary hypertension	15
Asthma	84	Non-asthmatics	63
Cardiomyopathy	47	Non-cardiomyopathy	38
Heart failure	36	Non-failing heart	19
Aortic stenosis	27	No aortic stenosis	16
Coronary artery disease	29	No coronary artery disease	20
Obesity	44	Non-obese	30

## Data Availability

The original data presented in the study are openly available in Gene Expression Omnibus 2R (NCBI) interactive web tool (https://www.ncbi.nlm.nih.gov/geo/query/acc.cgi), and iLNCS interactive web platform for the analysis of the Library of Integrated Network-Based Cellular Signatures (LINCS) (https://www.ilincs.org/ilincs/).

## Appendix A

DataSeries examined in this analysis are as follows, with GSEXXXX referring to NCBI-GEO and E-XXXX-XXXX referring to EMBL-EBI:
Pulmonary Tissues: (1, GSE144274; 2, GSE236251; 3, GSE130391; 4, GSE2125; 5, GSE2125; 6, E-GEOD-63142; 7, E-GEOD-19187; 8, E-GEOD-19187; 9, GSE3320; 10, GSE21411; 11, GSE21411);
Cardiac Tissues: (1, GSE1145; 2, GSE67492; 3, GSE3586; 4, GSE120836; 5, GSE42955; 6, GSE192886; 7, GSE161472; 8, GSE26887; 9, GSE10161; 10, GSE230585; 11, GSE235995; 12, GSE242046);
Renal Tissues: (1, GSE104948; 2, GSE28345; 3, GSE104954; 4, GSE28360; 5, GSE104948; 6, GSE30528; 7, GSE104954; 8, GSE30529; 9, GSE66494; 10, GSE20602; 11, GSE46699; 12, GSE46699; 13, GSE46699; 14, GSE46699);
Blood and PBMCs: (1, GSE24752; 2, GSE24760; 3, GSE75360; 4, GSE75360; 4, GSE48424; 5, GSE69601; 6, E-TABM-666; 7, GSE9006; 8, GSE55098; 9, GSE9006; 10, GSE23561; 11, GSE131793; 12, GSE42057; 13, GSE59019; 14, GSE16032; 15, GSE15072; 16, GSE37171; 17, GSE15072; 18, GSE37171; 19, GSE87493; 20, GSE69039; 21, GSE32575; 22, GSE23561; 23, GSE66360).
